# Plants as Biofactories to Produce Food, Medicines, and Materials for a True Green Revolution

**DOI:** 10.3390/ijms23105827

**Published:** 2022-05-23

**Authors:** Armando Zarrelli

**Affiliations:** Department of Chemical Sciences, University of Naples Federico II, 80126 Naples, Italy; zarrelli@unina.it

A proper diet is the basis of a healthy life; according to the WHO, approximately 1/3 of cardiovascular diseases and cancers can be avoided with a balanced and healthy diet [1]. For example, numerous in vitro and in vivo studies have shown that the consumption of black chokeberry fruits (*Aronia melanocarpa* L.) is associated with significant improvements in hypertension, oxidation of LDL (low-density lipoprotein), lipid peroxidation, total plasma antioxidant capacity, and dyslipidemia [2].

Plants have always been sources of useful molecules for humans. In the history of pharmacology, so-called medicinal plants have been highlighted for their fundamental role as sources of therapeutic molecules. It is no coincidence that 5% of drugs based on small molecules are natural products (~850 of those currently on the market), 27% are derived from natural products, and 31% are synthetic products inspired by natural products (the remaining 37% are purely synthetic products) [3]. These data indicate that molecules of natural origin have physicochemical structures suitable for interacting with cellular targets of pharmacological interest. Known examples include Vincristine, an alkaloid extracted from the leaves of *Catharanthus vinca* with highly effective anticancer activity, and Artemisinin, a sesquiterpene extracted from the leaves of *Artemisia annua*, considered the most effective antimalarial compound against resistant strains of Plasmodium. Therefore, plants still represent an important and, in some cases, irreplaceable source for the identification of lead compounds and for the design of new drugs.

Let us consider rhubarb—a well-known plant around the world that includes about 60 species of the genus Rheum. One of the representative plants is *Rheum palmatum*, which is prescribed in the Korean and Chinese pharmacopoeia for its pharmacological potential. The screening of the ethanolic extract led to the isolation of *Chrysophanol* 8-*O*-*glucoside*, of which biological assays have provided experimental evidence of protective effects against liver damage, as well as antifibrotic effects [4].

In general, natural pigments such as carotenoids, flavonoids, and anthocyanidins not only determine the attractive color of fruits but are also essential secondary metabolites that play multiple roles in the entire life cycle of plants and are characterized by powerful antioxidant activity. After decades of research and development, the multiple benefits of these natural pigments for human health have been explored and recognized and have shown brilliant potential for their applications in the food, medical, and cosmetic sectors [5].

The flavonoid quercetin has been shown to prevent nephrotoxicity in animal models and in a clinical trial, and it is therefore a very promising candidate for development. It is true that its clinical application is hampered by its low solubility, but a micellar formulation could help to overcome this problem by enhancing its kinetic and biopharmaceutical properties while employing lower dosages and administration routes oriented to its clinical use [6].

In general, however, secondary metabolites in plants are synthesized at very low concentrations—typically less than 1% of the dry weight of the plant itself. They are produced in such low quantities (e.g., 1 g of vincristine is obtained from 5 quintals of plant material) that the costs for their extraction and purification are very high, and in any case, the quantities available are often insufficient for the characterization of their activity. This easily explains the need to optimize synthetic production by lowering costs, with strong problems related to extensive chirality.

To meet the ever-increasing demand for new drugs in ever-greater quantities, researchers are learning to use plants as small drug “factories”, reducing production costs and guaranteeing even higher safety standards. This approach is called Plant Molecular Farming (Figure 1). In the past 20 years, genetic engineering has made it possible to generate transgenic plant lines with higher concentrations of the desired chemicals, with non-negligible implications for the sustainability of the product on the whole, especially in economic and environmental terms [7].

While plants are needed by humans, to whom they provide food and medicines, they can also help the entire planet Earth, which is experiencing a profound environmental crisis, to overcome challenges in specific sectors, such as that linked to pollution from plastic. In fact, some practical applications of these polymers are fundamental and indispensable, such as their applications in medical devices, the end-of-life management of which unfortunately remains problematic. This problem requires a redesign of the entire supply chain, opening the door to alternatives to fossil plastics, such as new biomaterials that reduce pollution upstream.

For example, it is now possible to prepare sustainable and degradable elastic mixtures from the copolymerization of natural rubber and polylactic acid, reinforced with linen fiber and montmorillonite. The obtained product shows significant improvements in plasticization, tensile strength, elasticity, and aging. Additionally, mold tests have revealed that adding plant-based fiber could significantly increase the biodeterioration potential, allowing for faster and more efficient growth of microorganisms. Therefore, such materials can become competitive and environmentally friendly alternatives to commonly used petroleum-based polymeric products [8].

Ultimately, the diversity of molecules that can be obtained from plants is still largely unexplored, but the demand for larger quantities of them will find an effective and sustainable response in advanced biotechnologies, accelerating the time of arrival of new or well-known products to the market, and opening the sector to hitherto never imagined new solutions. Plants therefore prove to be competitive in terms of costs and product quality with respect to traditional systems, allowing for greater accessibility to treatments in both general and particularly disadvantaged contexts. In the next two years, the value of the bioactive molecules sector is expected to exceed USD 100 billion. The Green Revolution is also emerging in this sector, and plants are taking off in applications where the advantages could make plant biofactories the preferred choice.

## Figures and Tables

**Figure 1:**
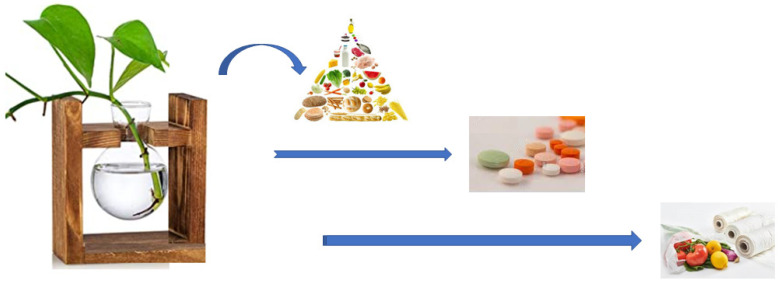
Plants as the primary source of everything we need.
